# EHMTI-0192. Onabotulinumtoxina prophylaxis in chronic migraine utilization and patient characteristics: observational study in the European Union

**DOI:** 10.1186/1129-2377-15-S1-G24

**Published:** 2014-09-18

**Authors:** M Matharu, J Pascual, I Nilsson Remahl, D Odom, L Gutierrez, E Andrews, J Largent, C Johannes

**Affiliations:** 1 Headache Group, Institute of Neurology National Hospital for Neurology and Neurosurgery, London, UK; 2 Neurociencias, Hospital Universitario Central de Asturias (HUCA) and INEUROPA, Oviedo, Spain; 3 Department of Clinical Neuroscience Division of Neurology, Karolinska Institute Karolinska University Hospital Huddinge, Stockholm, Sweden; 4 Biostatistics, RTI Health Solutions, Research Triangle Park, USA; 5 Epidemiology, RTI Health Solutions, Barcelona, Spain; 6 Pharmacoepidemiology and Risk Management, RTI Health Solutions, Research Triangle Park, USA; 7 Global Safety and Epidemiology, Allergan Inc., Irvine, USA; 8 Epidemiology, RTI Health Solutions, Waltham, USA

## Objective

Evaluate onabotulinumtoxinA utilization for chronic migraine (CM) headache prophylaxis in clinical practice and describe practice, physician, and patient characteristics.

## Methods

This is a prospective, observational, multinational European post-authorization study among adults treated with onabotulinumtoxinA for CM headache prophylaxis. Participating physicians recruited patients receiving treatment during routine care (September 2011-February 2014). The study aims to describe usage patterns and safety profile of onabotulinumtoxinA. Baseline characteristics and utilization at first injection on study are summarized using descriptive statistics.

## Results

As of January 1, 2014, 1141 patients treated by 86 physicians (81% neurologists) at 59 practices across UK, Germany, Sweden, and Spain were evaluated. The CM patient profile was generally comparable between countries. The average age was 46.4 years (range=19-79), 84% were female, 86% had a CM diagnosis recorded, and average number of headache-free days per usual month was 7.8 (SD=6.9). The most common medical conditions were depression (30%) and neck pain (27%). Most patients (86%) were using acute headache medications at baseline and 51% other preventive medications; half had received onabotulinumtoxinA for CM before the study. In the first treatment on study, the number of injections (median=31) above muscle areas was similar between countries. The majority of patients (78%, range=57%-95%) received a total dose between 150 and 200 units.

## Conclusions

Data from this study indicates that onabotulinumtoxinA is used in the appropriate patient population. Although further analysis is required, utilization of onabotulinumtoxinA generally appears to be consistent with aspects of the published PREEMPT injection paradigm.

